# Supplementary material for: Comparison of therapeutic effects between big-bubble deep anterior lamellar keratoplasty and penetrating keratoplasty for medically unresponsive Acanthamoeba keratitis
Source: BMC Infect Dis. 2024 Mar 4;24:276. doi: 10.1186/s12879-024-09147-w (PMC10910827; doi:10.1186/s12879-024-09147-w)
Supplement: Supplementary file 1 — Supplementary Material 1 [file 12879_2024_9147_MOESM1_ESM.docx]

**Comparison of therapeutic effects between big-bubble deep anterior lamellar keratoplasty and penetrating keratoplasty for medically unresponsive *Acanthamoeba* keratitis**

Xiaolin Qi,^1^ Huilin Mao,^1^ Jinhui Liu,^1^ Yanling Dong,^2^ Man Du,^1^ Ting Liu,^2^ Ting Zhang,^2^ Xiuhai Lu,^1^ Hua Gao^1^

^1^Eye Institute of Shandong First Medical University, Eye Hospital of Shandong First Medical University (Shandong Eye Hospital), State Key Laboratory Cultivation Base, Shandong Provincial Key Laboratory of Ophthalmology, School of Ophthalmology, Shandong First Medical University

^2^Eye Institute of Shandong First Medical University, Qingdao Eye Hospital of Shandong First Medical University, State Key Laboratory Cultivation Base, Shandong Provincial Key Laboratory of Ophthalmology, School of Ophthalmology, Shandong First Medical University

**Correspondence:** Prof. Hua Gao, Eye Hospital of Shandong First Medical University (Shandong Eye Hospital), 372 Jingsi Road, Jinan 250021, China. Phone: 86-531-81276112, fax: 86-531-81276090, E-mail: hgao@sdfmu.edu.cn

**Keywords:** *Acanthamoeba* keratitis, big-bubble deep anterior lamellar keratoplasty, penetrating keratoplasty, postoperative recurrence, immune rejection

**Abstract**

**Purpose:** To compare the outcomes of big-bubble deep anterior lamellar keratoplasty (BB-DALK) and penetrating keratoplasty (PKP) in the management of medically unresponsive *Acanthamoeba* keratitis (AK).

**Methods:** This retrospective study included 27 eyes of BB-DALK and 24 eyes of PKP from a tertiary ophthalmology care centre. Glucocorticoid eye drops were subsequently added to the treatment plan 2 months postoperatively based on the evaluation using confocal laser scanning microscopy. The clinical presentations, best-corrected visual acuity (BCVA), postoperative refractive outcomes, graft survival, and *Acanthamoeba* recurrence were analyzed.

**Results:** The AK patients included in the study were in stage 2 or stage 3, and the percentage of patients in stage 3 was higher in the PKP group (*P*=0.003). Clinical presentations were mainly corneal ulcers and ring infiltrates, and endothelial plaques, hypopyon, uveitis and glaucoma were more common in the PKP group (*P*=0.007). The BCVA and the graft survival rate showed no statistically significant differences between the two groups at 1 year after surgery. However, 3 years postoperatively, the BCVA of 0.71±0.64 logMAR, the graft survival rate of 89.5%, and the endothelial cell density of 1899±125 cells per square millimeter in the BB-DALK group were significantly better than those of the PKP group (*P*=0.010, 0.046, and 0.032, respectively). 3 eyes (11.1%) in the BB-DALK group and 2 eyes (8.3%) in the PKP group experienced *Acanthamoeba* recurrence, but the rates showed no statistically significant difference between the two groups (*P*=1.000). In the PKP group, immune rejection and elevated intraocular pressure were observed in 5 and 6 eyes, respectively.

**Conclusion**: Corneal transplantation is recommended for AK patients unresponsive to antiamoebic agents. The visual acuity and graft survival can be maintained after BB-DALK surgery. *Acanthamoeba* recurrence is not related to the surgical approach performed, whereas complete dissection of the infected corneal stroma and delayed prescribing of glucocorticoid eye drops were important to prevent recurrence.

**Introduction**

*Acanthamoeba* keratitis (AK) is an uncommon but sight-threatening corneal infection caused by several amoebas of the genus *Acanthamoeba*. In developed countries, contact lens use is considered as the predominant risk factor for AK^1-4^, whereas in China, the most common risk factor identified was ocular trauma, followed by exposure to contaminated water^5-7^. In consequence, the majority of AK patients were farmers, but they had poor awareness of medical treatment, which easily delayed the illness. Moreover, few topical antiamoebic agents are available because of their poor corneal penetration and topical toxicity. Doctors must therefore remove the pathogens via corneal transplantation. Penetrating keratoplasty (PKP) is a full-thickness transplant procedure, performed to replace the damaged or diseased cornea with healthy corneal tissue. The donor corneal tissue used in PKP procedures is required to be fresh, but even so, postoperative endothelial immune rejection may still occur. For another widely-performed transplant procedure, lamellar keratoplasty, higher rates of postoperative recurrence have been noted and the residual recipient stroma may have an adverse influence on visual outcomes. To get better surgical results, ophthalmologists have made efforts to improve surgical skills. Big-bubble technique (BB-DALK) has been frequently performed in deep anterior lamellar keratoplasty procedures, after which the postoperative recurrence rate is relatively lower while the visual outcomes are better owing to the removal of the corneal stroma down to Descemet’s membrane (DM)^8,9^. However, few studies have compared the visual outcomes and complications in patients with AK after BB-DALK or PKP. In order to address this, we compared the postoperative visual acuity, graft survival, *Acanthamoeba* recurrence, and immune rejection in patients with medically unresponsive AK after BB-DALK or PKP.

**Patients and Methods**

**Patients**

This retrospective study was approved by the Institutional Review Board of Eye Hospital of Shandong First Medical University. This study was conducted in compliance with the tenets of the Declaration of Helsinki.

A consecutive series of 51 eyes (51 patients) was diagnosed with AK at the Eye Hospital of Shandong First Medical University from August 2013 to August 2020. AK was diagnosed as 1 or more of the following: (1) positive corneal scraping; (2) positive confocal microscopy; (3) positive culture on corneal scraping; and (4) identification of cysts or trophozoites on biopsy specimens. Patient data included age, gender, profession, predisposing risk factors, clinical presentation, and surgical treatment were collected. The clinical presentation was divided into three stages according to the method reported by Robaei et al^10,11^.

**Medical and surgical treatment**

Following a diagnosis of AK, the patients were administered topically chlorhexidine 0.02% every 30 minutes, polyhexamethylene biguanide (PHMB) 0.02% every 30 minutes, and metronidazole every 1 h. Therapeutic keratoplasty was considered if the corneal inflammatory reaction was not controlled after 2 weeks of antiamoebic drug therapy. The clinical presentations included: corneal stromal edema and infiltrate gradually worsened; corneal ulcers did not heal, but had a tendency to expand, and even with a risk of perforation; and hypopyon gradually increased. DALK surgery was considered in patients with infection or infiltrates greater than 4/5ths of the corneal thickness, but not involved the whole layer, as observed by slit-lamp microscopy, anterior segment optical coherence tomography (As-OCT), and after DM was exposed. For patients detected with endothelial plaque during BB-DALK, or diagnosed with perforation, PK was performed instead.

**Surgical procedures**

All surgeries were performed by a single surgeon (H.G.). In patients with combined hypopyon, a lateral incision was first made at the corneal limbus, and the anterior aqueous humor was injected into the anterior chamber to flush the hypopyon away. The size of the Hessburg-Barron vacuum trephine (Katena, Denville, New Jersey, USA) was selected according to the scope of the corneal ulcer; its diameter was 1.5 mm greater than that of the corneal ring infiltrate. Approximately 300μm of corneal thickness was dissected using a corneal trephine. After a large bubble was injected into the posterior stroma, the diseased stroma was cut oﬀ using a 45° blade to bare the DM. After DM was exposed, the recipient bed was assessed under the surgical microscope. If the recipient bed was clear, and no endothelial plaques were visible, DALK was continued. In case of infiltration of the residual corneal stoma, or endothelial plaques in DM, PK was conducted instead^12,13^. A corneal graft with a diameter of 0.25 mm more than the recipient bed was sutured to the recipient. After surgery, the diseased corneal tissue was sent for fungal pathogen culture and pathological examination.

**Postoperative treatment**

The patients were treated with combination topical antiamoebic agents continually. 0.1% tacrolimus eye drops were prescribed to all patients 4 times a day for 2 months. No systemic or local glucocorticoid medication was applied within 2 months after surgery, and glucocorticoid eye drops were gradually applied after 2 months based on the scanning by confocal laser scanning microscopy. If no *Acanthamoeba* cysts were observed in the graft and recipient bed, 0.02% fluorometholone eye drops were first given twice daily, and the site was re-examined by confocal laser scanning microscopy after 2 weeks of application. Tobramycin dexamethasone eye ointment was applied once every night if no *Acanthamoeba* recurrence was observed. Subsequently, all the patients were observed monthly for 6 months. The dosage of glucocorticoid eye drops was adjusted if the patients’ condition remained stable and there were no signs of *Acanthamoeba* recurrence. Tacrolimus was discontinued after glucocorticoid addition in patients with BB-DALK.

The best-corrected visual acuity (BCVA), intraocular pressure, postoperative refractive outcomes, graft survival, endothelial cell density, perioperative and postoperative complications were evaluated at 1 and 3 years after surgery. The BCVA was recorded by the Snellen chart and converted logarithm of minimal angle of resolution (LogMAR) units for statistical analysis according to the methods of Moussa et al^14^.

In addition, corneal buttons obtained during repeated keratoplasty from *Acanthamoeba* recurrence patients were stained with hematoxylin-eosin (HE) stain to observe distribution of *Acanthamoeba* cysts.

**Statistical analysis**

Statistical analyses were performed using SPSS 24.0 (SPSS, Chicago, Illinois, USA). A P-value of < 0.05 was considered statistically significant. Baseline characteristics are presented as Mean±SD or Median (IQ1,IQ3) for continuous variables and n(%) for categorical variables. The main demographics, preoperative differences and postoperative refractive status between groups were compared with the Wilcoxon test, t test, chi-square test or Fisher’s exact test. Survival analysis was performed using Kaplan-Meier analysis method and log-rank test.

**Results**

**Patient information**

The patients were divided into two groups in accordance with their surgical treatment: 27 eyes in the BB-DALK group and 24 eyes in the PKP group. The mean follow-up period of BB-DALK group was 26.78±21.11 months (range, 0-78 months), whereas 27.29±21.23 months (range, 0-79 months) in the PKP group, the comparisons showed no significant differences (t=-0.087*, P*=0.931). The demographics and preoperative characteristics of the two groups were comparable, and the intergroup comparisons showed no significant differences (Table 1).

**Table 1.** **Preoperative characteristics of patients in BB-DALK group and PKP**

**group**

| **Characteristics** | **BB-DALK group** | **PKP group** | ***P*** |
| --- | --- | --- | --- |
| **No. Eyes** | 27 | 24 |  |
| **Age, y (**Mean±SD) | 44.56±15.5 | 48.79±15.7 | 0.339 |
| **Gender** n(%)  Male  Female | 17(63.0)  10(37.0) | 11(45.8)  13(54.2) | 0.220 |
| **Profession** n(%)  Farmer  Worker  Professional technicians  Student  Other | 7(25.9)  1(3.7)  11(40.7)  3(11.1)  5(18.5) | 7(29.2)  2(8.3)  5(20.8)  2(8.3)  8(33.3) | 0.494 |
| **Onset of symptoms to keratoplasty, d**  Median (Range) | 43(18-96) | 78.5(20-313) | 0.011*** |
| **Causes** n (%)  Contact lens wear  Contaminated water  Plant trauma  Foreign body  Unknown reason | 3(11.1)  9(33.3)  6(22.2)  5(18.5)  4(14.8) | 2(8.3)  7((29.2)  5(20.8)  6(25)  4(16.7) | 0.991 |
| **Treatment prior to diagnosis of AK** n(%)  Fungal keratitis  HSV keratitis  Topical corticosteroid use | 2(7.4)  11(40.7)  12(44.4) | 2((8.3)  6(25.0)  7(29.2) | 0.902 |
| **Preoperative LogMAR BCVA**  Mean (SD) | 1.95±0.61 | 2.93±0.39 | 0.039*** |
| **Clinical presentations at time of keratoplasty** n(%)  Ring infiltrate  Endothelial plaques  Hypopyon  Uveitis  Glaucoma | 8(29.6)  2(7.4)  4(14.8)  2(7.4)  1(3.7) | 17(70.8)  18(75)  12(50)  18(75)  9(37.5) | 0.024*** |
| **Disease stage at presentation** n(%)  Stage 2  Stage 3 | 19(70.4)  8(29.6) | 7(29.2)  17((70.8) | 0.003*** |

**Preoperative clinical presentations**

Sudden pain with rubbing, photophobia, tearing, and decreased visual acuity in the keratoplasty was 43.0 days (range, 18-96 days) in the BB-DALK group, whereas 78.5 days (range, 20-313 days) in the PKP group (*P*=0.011).

The preoperative BCVA (logMAR) was 2.93±0.39 in the PKP group (Figure 1), which showed statistically significant worse than that in the BB-DALK group (Figure 2), which was 1.95±0.61 (*P*=0.039).

**Figure 1**


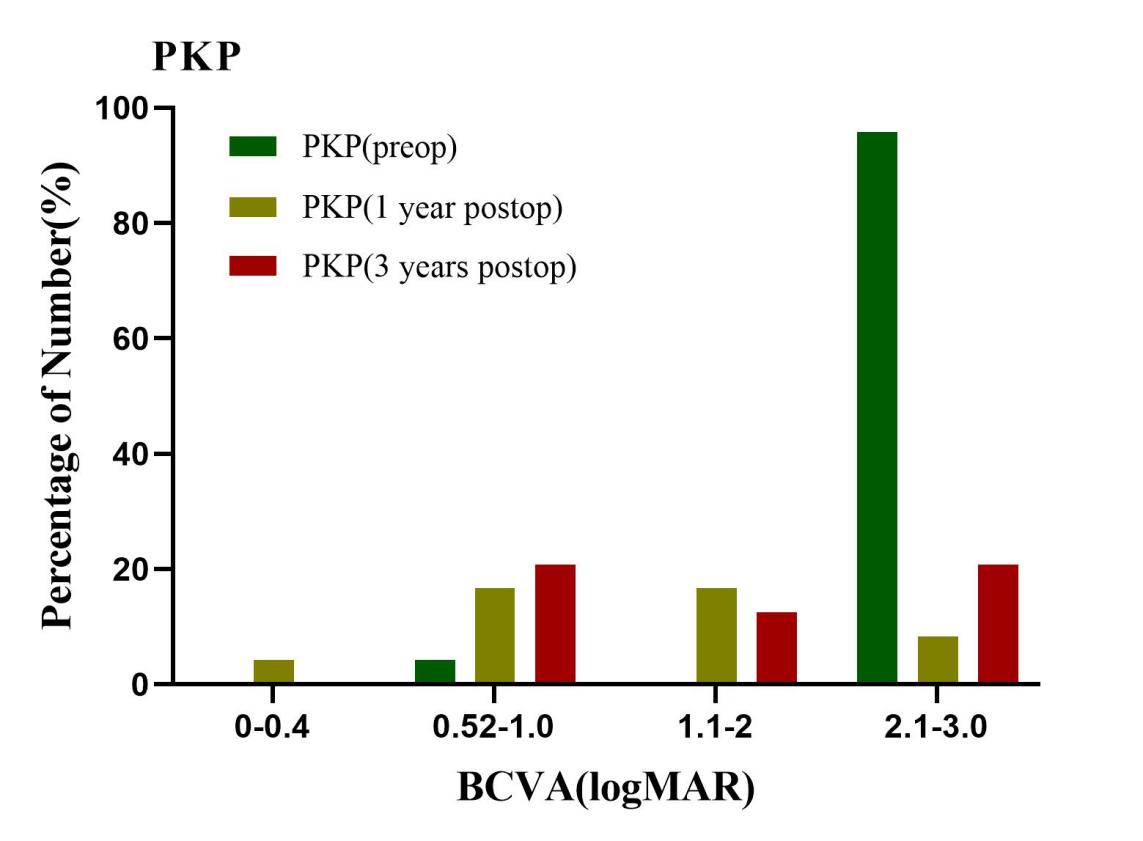


**Figure 2**


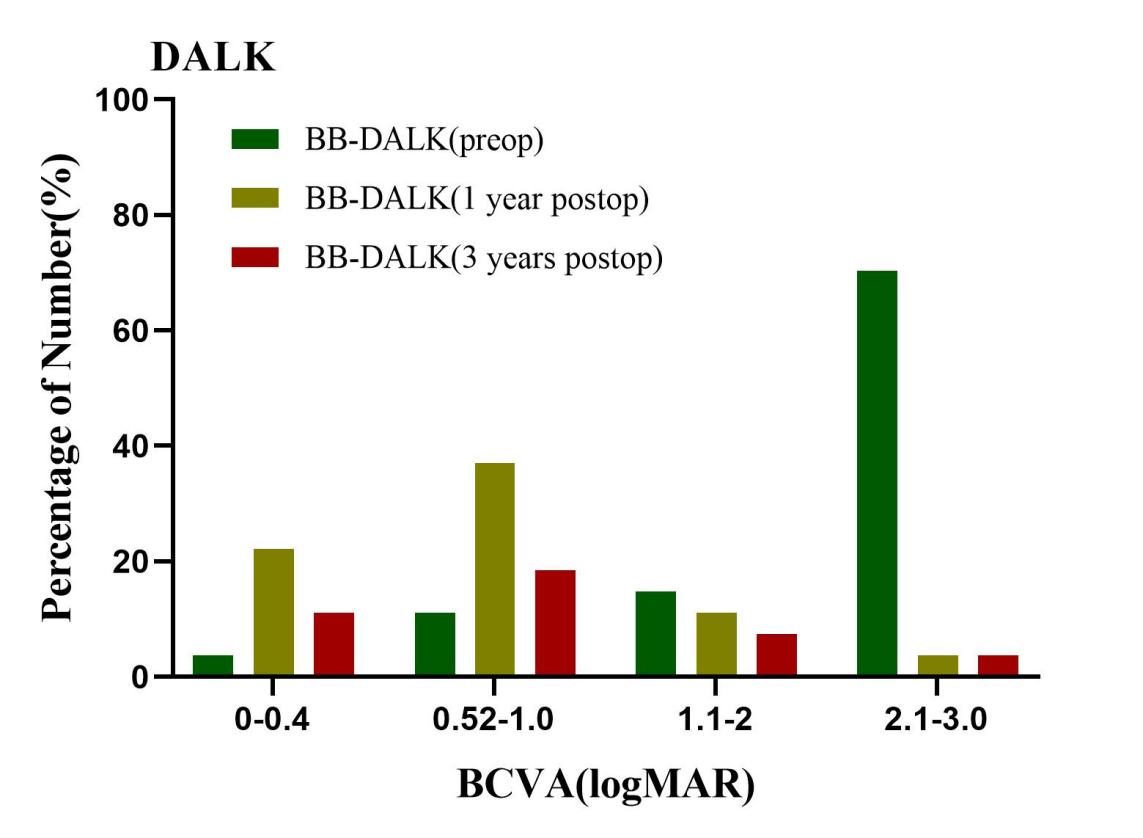


In our study, the disease stages at presentation in the two groups were stage 2 and stage 3, with the percentage of stage 2 higher in the BB-DALK group, whereas the percentage of stage 3 higher in the PKP group (*P*=0.003). The clinical presentations (Figure 3) were mainly corneal ulcers and ring infiltrate, and the ring infiltrate, endothelial plaque, hypopyon, uveitis, and glaucoma were more common in the PKP group (*P*=0.024, Table 1).

*Acanthamoeba* cysts were found in 46 cases (90.2%) on examination of corneal scrapings and 31 cases (60.8%) examined by confocal microscopy. A total of 38 specimens (74.5%) had positive culture results for cysts.

**Figure 3**


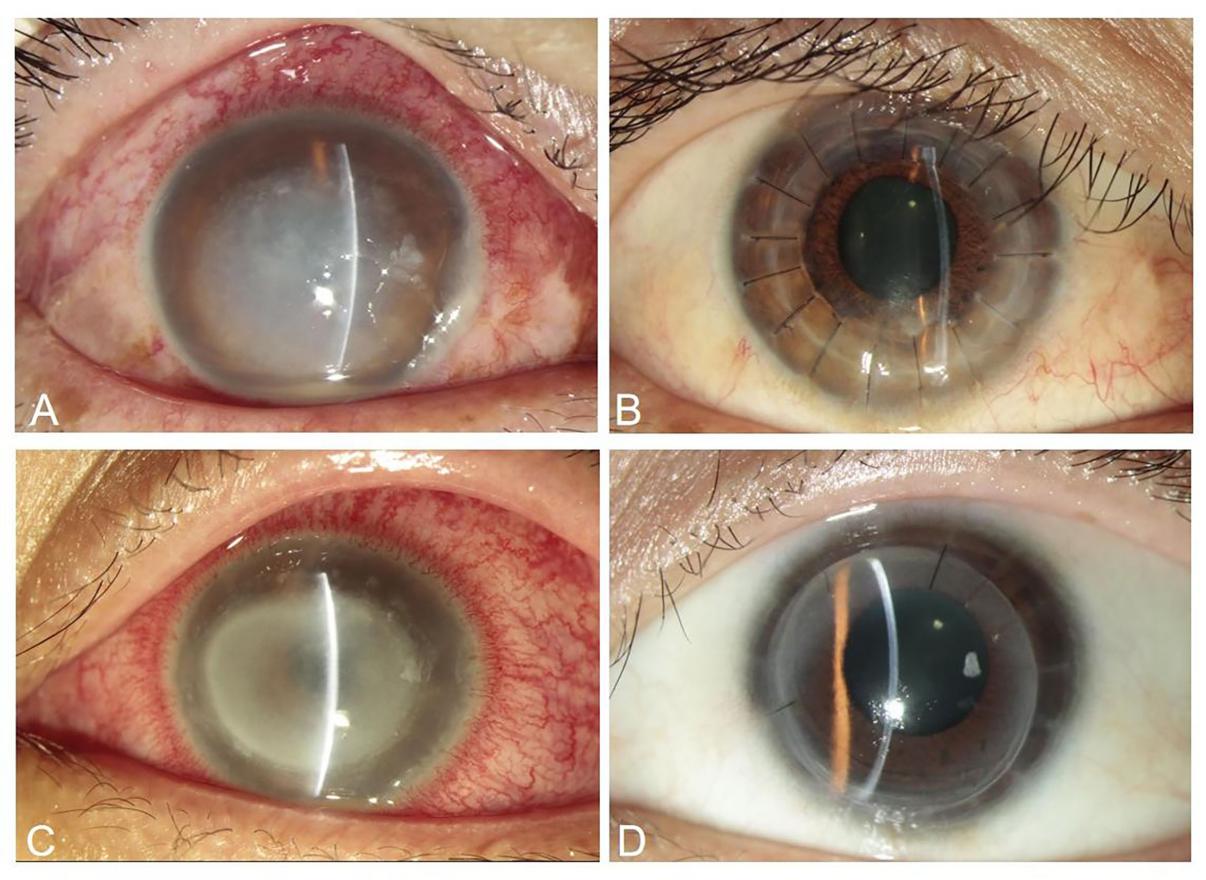


**Postoperative VA and refractive** **outcomes**

At 1 year after surgery, the BCVA (logMAR) was 0.71±0.59 in the BB-DALK group (Figure 2), which showed no differences from that in the PKP group (Figure 1), which was 0.79±0.66 (*P*=0.144). The curvature was 44.51±2.94D and 45.62±2.97D in the BB-DALK and PKP groups, respectively (*P*=0.214). The astigmatism and equivalent spherical radius in the two groups showed no statistically significant differences (*P*>0.05, Table 2).

At 3 years after surgery, the BCVA (logMAR) was 0.71±0.64 in the BB-DALK group (Figure 2), which showed statistically significant better than that in the PKP group (Figure 1), which was 0.93±0.76 (*P*=0.010). The curvature was 44.99±2.42D and 46.03±2.38D in the BB-DALK and PKP groups, respectively (*P*=0.861). The astigmatism and equivalent spherical radius in the two groups showed no statistically significant differences (*P*>0.05, Table 2).

**Table 2. Postoperative Refractive Status of the BB-DALK group and PKP group**

|  | **1 year after surgery** | | | **3 years after surgery** | | |
| --- | --- | --- | --- | --- | --- | --- |
|  | **BB-DALK group** | **PKP group** | ***P* value** | **BB-DALK group** | **PKPgroup** | ***P* value** |
| **BCVA (logMAR)** | 0.71±0.59 | 0.79±0.66 | 0.144 | 0.71±0.64 | 0.93±0.76 | 0.010* |
| **Corneal Curvature** | 44.51±2.94 | 45.62±2.97 | 0.214 | 44.99±2.42 | 46.03±2.38 | 0.861 |
| **Astigmatism** | -3.06±3.88 | -4.08±2.15 | 0.315 | -3.51±3.34 | -4.83±1.75 | 0.091 |
| **Equivalent Spherical Radius** | -1.72±2.74 | -2.70±2.21 | 0.569 | -1.83±2.89 | -2.87±3.03 | 0.179 |
| **Endothelial Cell Count** | 1985±112 | 1895±232 | 0.088 | 1899±125 | 1608±231 | 0.032* |
| BB-DALK =Big-Bubble Deep Anterior Lamellar Keratoplasty; BCVA=Best-Corrected Visual Acuity | | | | | | |

**Graft Survival**

The graft survival rate was 95.5% and 88.9% at 1 year after surgery in the BB-DALK and PKP group, respectively. Kaplan-Meier analysis and log rank tests showed no significant differences between the two groups (*P*=0.467).

The graft survival rate was 89.5% and 61.1% at 3 years after surgery in the BB-DALK and PKP group, respectively. Kaplan-Meier analysis and log rank tests showed statistically significant differences between the two groups (*P*=0.046, Figure 4).

**Figure 4**


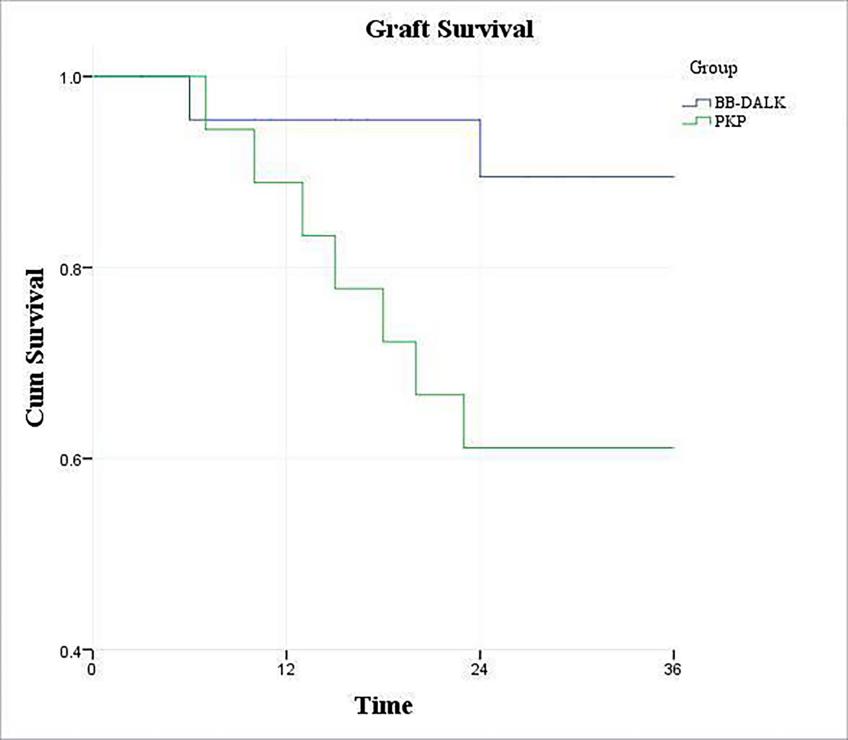


**Loss of corneal endothelial cells**

The endothelial cell density was 1985±112cells per square millimeter at 1 year after surgery in the BB-DALK group, with no statistically significant differences compared with that in the PKP group, which was 1895±232cells per square millimeter (*P*=0.088, Table 2).

The endothelial cell density was 1899±125cells per square millimeter at 3 years after surgery in the BB-DALK group, with statistically significant differences compared with that in the PKP group, which was 1608±231 cells per square millimeter (*P*=0.032, Table 2).

***Acanthamoeba* recurrence after keratoplasty**

The diagnostic methods for the detection of *Acanthamoeb*a recurrence were according to the reference of Zhang et al^7^. 5 eyes (9.8%) including 3 eyes (11.1%) in the BB-DALK group and 2 eyes (8.3%) in the PKP group experienced *Acanthamoeba* recurrence after keratoplasty. The rates of recurrence showed no difference between the two groups (*P*=1.000).

2 eyes experienced *Acanthamoeba* recurrence in the graft-host junction in the PKP group. The recipient bed was stained with HE at the recurrence site, which revealed scattered *Acanthamoeba* cysts at the cut edge of the recipient bed. This indicated that the trephined range during corneal transplantation did not cover all the *Acanthamoeba* infected stroma, which led to the postoperative recurrence (Figure 5).

**Figure 5**


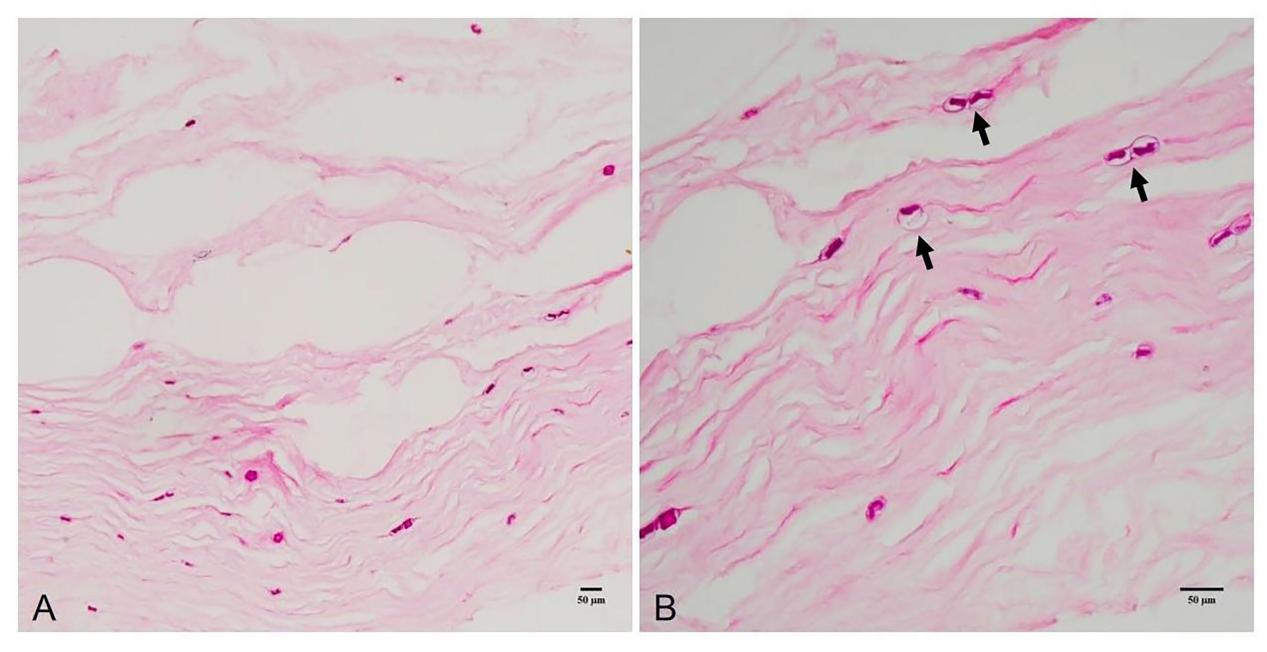


3 eyes (11.1%) experienced *Acanthamoeba* recurrence in the recipient bed in the BB-DALK group. The HE stain of the corneal buttons obtained during repeated keratoplasty revealed scattered distribution of *Acanthamoeba* cysts at the graft-recipient bed junction, indicating that the depth of dissection was not sufficient to remove all the *Acanthamoeba* infected stroma during corneal transplantation (Figure 6).

**Figure 6**


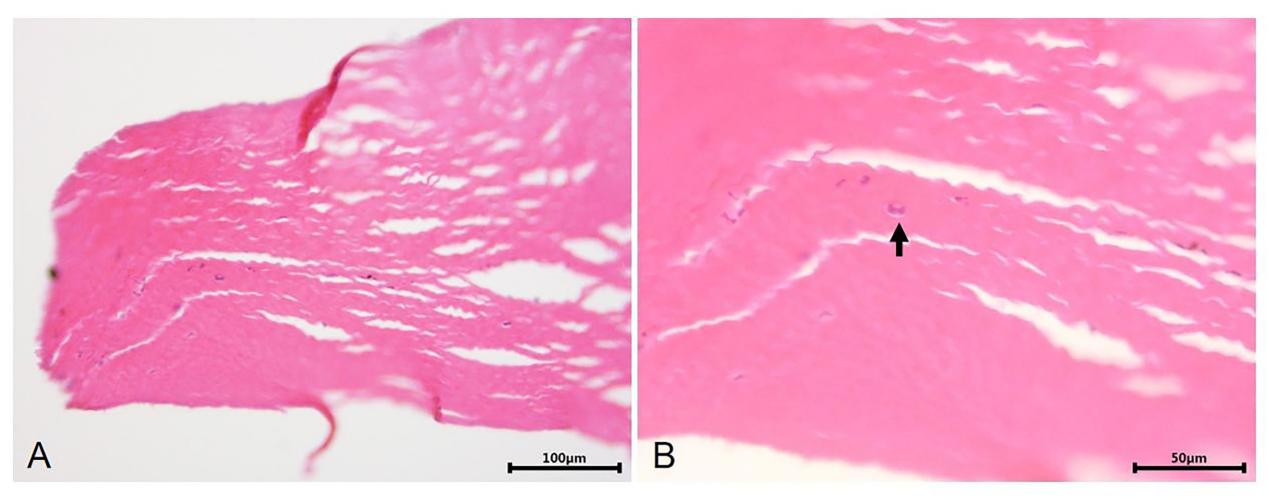


**Intraoperative and postoperative complications**

Intraoperative microperforation of the DM occurred in 4 eyes during stromal dissection in the BB-DALK group. These four patients still received DALK with an air bubble injected into the anterior chamber. No cases of hyphema, iris damage, and lens dislocation were observed during the surgery in the PKP group.

In the PKP group, immune rejection was observed in 5 eyes, the rate of rejection episodes was 20.8%. In addition, elevation of intraocular pressure was detected in 6 eyes preoperatively. The intraocular pressure returned to normal after antiglaucoma medication in 4 eyes within 1 month after surgery. The other two patients refused to undertake trabecular surgery, the intraocular pressure was controlled at 22-28mmHg.

Finally, complicated cataract was observed in 3 eyes.

In the BB-DALK group, no immune rejection was detected during the follow-up. However, elevation of intraocular pressure was detected in 1 eye preoperatively, which was controlled with local medical therapy within 1 month after surgery.

**Discussion**

AK is an uncommon but sight-threatening corneal infection. Prognosis is often poor because of a significant delay in diagnosis and a lack of effective medical treatment^15,16^. Regarding the timing of corneal transplantation, studies in developed countries recommend a continuous medical treatment for more than 3 months and optical keratoplasty (OKP) when corneal inflammation subsides and stromal scar forms^11^. This can reduce the recurrence of infection and the need for repeated corneal transplantation. However, AK patients in China generally experience chronic disease, associated with a high degree of infection. These conditions are often combined with intraocular infections, such as hypopyon and uveitis^5-7^. In this study, all AK patients were in stages 2 and 3, and the clinical presentations of corneal ulcers and ring infiltrate were more common in the PKP group (*P=*0.024). An important feature of the ring infiltrate was its blurred boundary, which did not allow clear distinction of the extent of *Acanthamoeba* infection. Tu et al showed that the presence of ring infiltrate at diagnosis was independently associated with worse visual outcomes^17^. However, it is difficult to control such a serious infection with clinically applied antiamoebic drugs. As the infection gradually worsens to involve the limbus cornea or the whole layer leading to perforation, and a large-diameter PKP needs to be adopted, the risk of postoperative complications, such as non-healing of the graft epithelium and immune rejection, increases^18^. Therefore, corneal transplantation is an ideal treatment when there is no possibility of remission after treatment of medically refractory *Acanthamoeba* keratitis.

PKP and DALK are the current mainstream surgical approaches. In our study, we chose the appropriate surgical approach mainly based on the depth of the corneal ulcer. If the infection did not involve the DM, DALK was chosen, and when the infection involved the whole layer, PKP was selected^19^. We compared the differences in postoperative visual acuity, refractive status, and graft survival between the two surgical approaches. In the BB-DALK group, the postoperative BCVA (logMAR) was maintained at 0.7 at 1 and 3 years after surgery. Except for intraocular pressure elevation detected in one eye, no immune rejection or complicated cataracts were detected during the follow-up. Compared with the BB-DALK group, the PKP group demonstrated a significantly lower BCVA because of severe preoperative corneal infection and anterior chamber inflammation inevitably. In addition, immune rejection and elevated intraocular pressure were observed in 5 and 6 eyes, respectively, affecting the endothelial cells and graft survival. Three years postoperatively, the endothelial cell count and the graft survival rate of the PKP group were lower than those of the BB-DALK group, and these two indicators in patients with *Acanthamoeba* keratitis were also lower than those in patients with other corneal diseases including keratoconus^19^, corneal dystrophy^20^ and fungal keratitis^13,21^. Hence, if the whole corneal layer is not affected by *Acanthamoeba* infection, BB-DALK is recommended, as this can reduce the risk of postoperative complications, such as immune rejection and secondary glaucoma, and the visual acuity and graft survival rate can be maintained^22,23^.

*Acanthamoeba* recurrence after AK corneal transplantation is inevitable, the recurrence rate reported in the literature ranges from 3.6 to 86%^7,10,15^. 5 eyes (9.8%), including 3 eyes (11.1%) in the BB-DALK group and 2 eyes (8.3%) in the PKP group experienced in our study. The rates of recurrence showed no difference between the two groups (*P*=1.000). However, we consider that the reduced recurrence rate is not related to the surgical approach chosen. Thus, attention was given to the following three key points. First, trephines with an appropriate diameter were selected according to the extent of corneal ulcers during surgery (diameter of 1.5 mm greater than that of the ring infiltrate) to minimize the incidence of residual *Acanthamoeba* cysts.

In the PKP group, *Acanthamoeba* infection recurred in two eyes, particularly in the graft-host junction. Scattered *Acanthamoeba* cysts were observed at the cut edge of the recipient bed, indicating that the trephined range during corneal transplantation did not cover all the *Acanthamoeba*-infected tissues, which led to postoperative recurrence.

Second, care should be taken to determine whether the infection involves the whole corneal layer after exposure of the DM. In the BB-DALK group, HE staining revealed a scattered distribution of *Acanthamoeba* cysts at the graft-recipient bed junction in three eyes, indicating that the depth of dissection during BB-DALK was not sufficient to completely remove the *Acanthamoeba*-infected stroma. Therefore, if DM was clean and no infiltration or endothelial plaques were found, BB-DALK was performed, whereas PKP was performed instead. Accordingly, these principles were followed to guide corneal transplantation in patients with fungal corneal infections^12,13^. The recurrence rates were lower than those reported in previous studies, such as that of Ti et al^24^. Therefore, we consider that this method is worth applying.

Third, instead of applying topical glucocorticoid eye drops for 2 months postoperatively, immunosuppressant tacrolimus eye drops can be used as an alternative to prevent immune rejection. Glucocorticoid eye drops can reduce corneal inflammation, neovascularization, and treat scleritis, but Robaei et al concluded that corticosteroid use before diagnosis of AK was associated with an approximately 4-fold increased odds of a poorer visual outcome^10^. Moreover, corticosteroids have been shown in animal models in vivo to induce a 4- to 10-fold increase in the number of *Acanthamoeba* trophozoites^25^. Hence, FK506 is considered a better choice because it suppresses the expression of T cell-mediated lymphokines and the interleukin-2 receptor and the generation of cytotoxic T cells, which effectively prevents graft rejection and maintains the transparency of the graft. Likewise, Yuan et al demonstrated the lower rate of graft failure of PKP in 15 cases of AK was associated that FK506 was prescribed postoperatively whereas corticosteroids were avoided^26^.

There is a bias in the patient selection criteria in this retrospective study, which is inevitable. Firstly, there were differences in the clinical presentations and grading of the patients in the two groups before surgery. Secondly, the patients were not grouped based on randomization principles, but on intraoperative observation of whether the infection involved the full thickness.

Corneal infection and anterior chamber inflammation are serious issues in AK patients in China. Antiamoebic drugs are often ineffective; therefore, we advocate treatment with corneal transplantation as soon as possible. If the *Acanthamoeba* infection does not involve the whole layer of the cornea, BB-DALK is recommended to maintain the visual acuity and survival rate. To minimize residual *Acanthamoeba* cysts, the trephine range used during corneal implantation had a diameter of 1.5mm greater than that of the ring infiltrate. Another important measure for reducing the recurrence after corneal transplantation was avoiding the application of topical glucocorticoid drops within 2 months after surgery.

**Abbreviations**

BB-DALK big-bubble deep anterior lamellar keratoplasty

PKP penetrating keratoplasty

AK *Acanthamoeba* keratitis

BCVA best-corrected visual acuity

DM Descemet’s membrane

PHMB polyhexamethylene biguanide

As-OCT anterior segment optical coherence tomography

HE hematoxylin-eosin

OKP optical keratoplasty

**Acknowledgments**

The authors would like to thank Ms. Tong Liu from Eye Hospital of Shandong First Medical University for her editorial and linguistic assistance.

**Authors' contributions**

Design and conduct of the study (XQ, HG); Collection of the data (HM, YD, TZ); Management, analysis, and interpretation of the data (JL, TL); Preparation of the manuscript (XQ); Review and approval of the manuscript (HG). All authors have reviewed the final version of the manuscript and approve it for publication.

**Funding**

This study was supported by National Natural Science Foundation of China (82070923 and 81870639), Taishan Scholar Program (201812150), Academic Promotion Programme of Shandong First Medical University (2019RC009). The funding organization had no role in the design of the study and collection, analysis, and interpretation of data and in writing the manuscript.

**Availability of data and materials**

The datasets used and analysed during the current study available from the corresponding author on reasonable request.

**Declarations**

**Ethics approval and consent to participate**

All procedures performed in studies involving human participants were in accordance with the ethical standards of the ethical board committee of the Eye Hospital of Shandong First Medical University and with the 1964 Helsinki declaration and its later amendments or comparable ethical standard. All participants voluntarily agreed to participate in this study and all gave written informed consent.

**Consent for publication**

Not applicable.

**Competing interests**

The authors declare no competing interests.

**Reference**

1. Qian Y, Meisler DM, Langston RH, Jeng BH. Clinical experience with Acanthamoeba keratitis at the cole eye institute, 1999-2008. Cornea 2010;29(9):1016-1021.
2. Maycock NJ, Jayaswal R. Update on Acanthamoeba Keratitis: Diagnosis, Treatment, and Outcomes. Cornea 2016;35(5):713-720.
3. Lee MH, Abell RG, Mitra B, Ferdinands M, Vajpayee RB. Risk factors, demographics and clinical profile of Acanthamoeba keratitis in Melbourne: an 18-year retrospective study. Br J Ophthalmol 2018;102(5):687-691.
4. Carnt N, Robaei D, Minassian DC, Dart JKG. Acanthamoeba keratitis in 194 patients: risk factors for bad outcomes and severe inflammatory complications. Br J Ophthalmol 2018;102(10):1431-1435.
5. Sun X, Zhang Y, Li R, et al. Acanthamoeba keratitis: clinical characteristics and management. Ophthalmology 2006;113(3):412-416.
6. Jiang C, Sun X, Wang Z, Zhang Y. Acanthamoeba keratitis: clinical characteristics and management. Ocul Surf 2015;13(2):164-168.
7. Zhang T, Xie L, Dong Y, Cheng J. Therapeutic keratoplasty for severe Acanthamoeba keratitis: risk factors, clinical features, and outcomes of postoperative recurrence. Graefes Arch Clin Exp Ophthalmol 2022 Nov.
8. Gao H, Song P, Echegaray JJ, et al. Big bubble deep anterior lamellar keratoplasty for management of deep fungal keratitis. J Ophthalmol 2014;2014:209759.
9. Sabatino F, Sarnicola E, Sarnicola C, et al. Early deep anterior lamellar keratoplasty for fungal keratitis poorly responsive to medical treatment. Eye (Lond) 2017;31(12):1639-1646.
10. Robaei D, Carnt N, Minassian DC, Dart JK. The impact of topical corticosteroid use before diagnosis on the outcome of Acanthamoeba keratitis. Ophthalmology 2014;121(7):1383-1388.
11. Robaei D, Carnt N, Minassian DC, Dart JK. Therapeutic and optical keratoplasty in the management of Acanthamoeba keratitis: risk factors, outcomes, and summary of the literature. Ophthalmology 2015;122(1):17-24.
12. Qi X, Liu T, Du M, Gao H. Endothelial Plaques as Sign of Hyphae Infiltration of Descemet's Membrane in Fungal Keratitis. J Ophthalmol 2020;2020:6083854.
13. Chen X, Li X, Zhang X, et al. Comparison of complications and visual outcomes between big-bubble deep anterior lamellar keratoplasty and penetrating keratoplasty for fungal keratitis. Clin Exp Ophthalmol 2021;49(6):550-559.
14. Moussa G, Bassilious K, Mathews N. A novel excel sheet conversion tool from Snellen fraction to LogMAR including 'counting fingers', 'hand movement', 'light perception' and 'no light perception' and focused review of literature of low visual acuity reference values. Acta Ophthalmol 2021;99(6):e963-e965.
15. Kitzmann AS, Goins KM, Sutphin JE, Wagoner MD. Keratoplasty for treatment of Acanthamoeba keratitis. Ophthalmology 2009;116(5):864-869.
16. Papa V, Rama P, Radford C, Minassian DC, Dart JKG. Acanthamoeba keratitis therapy: time to cure and visual outcome analysis for different antiamoebic therapies in 227 cases. Br J Ophthalmol 2020;104(4):575-581.
17. Tu EY, Joslin CE, Sugar J, Shoff ME, Booton GC. Prognostic factors affecting visual outcome in Acanthamoeba keratitis. Ophthalmology 2008;115(11):1998-2003.
18. Roozbahani M, Hammersmith KM, Rapuano CJ, Nagra PK, Zhang Q. Therapeutic penetrating keratoplasty for acanthamoeba keratitis: a review of cases, complications and predictive factors. Int Ophthalmol 2019;39(12):2889-2896.
19. Bahar I, Kaiserman I, Srinivasan S, et al. Comparison of three different techniques of corneal transplantation for keratoconus. Am J Ophthalmol 2008;146(6):905-12.e1.
20. Reddy JC, Murthy SI, Vaddavalli PK, et al. Clinical outcomes and risk factors for graft failure after deep anterior lamellar keratoplasty and penetrating keratoplasty for macular corneal dystrophy. Cornea 2015;34(2):171-6.
21. Sabatino F, Sarnicola E, Sarnicola C, et al. Early deep anterior lamellar keratoplasty for fungal keratitis poorly responsive to medical treatment. Eye (Lond) 2017;31(12):1639-1646.
22. Sarnicola E, Sarnicola C, Sabatino F, et al. Early Deep Anterior Lamellar Keratoplasty (DALK) for Acanthamoeba Keratitis Poorly Responsive to Medical Treatment. Cornea 2016;35(1):1-5.
23. Bagga B, Garg P, Joseph J, Mohamed A, Kalra P. Outcome of therapeutic deep anterior lamellar keratoplasty in advanced Acanthamoeba keratitis. Indian J Ophthalmol 2020;68(3):442-446.
24. Ti SE, Scott JA, Janardhanan P, Tan DT. Therapeutic keratoplasty for advanced suppurative keratitis. Am J Ophthalmol 2007;143(5):755-762.
25. Carnt N, Robaei D, Watson SL, Minassian DC, Dart JK. The Impact of Topical Corticosteroids Used in Conjunction with Antiamoebic Therapy on the Outcome of Acanthamoeba Keratitis. Ophthalmology 2016 ;123(5):984-990.
26. Zhong J, Li X, Deng Y, et al. Associated factors, diagnosis and management of Acanthamoeba keratitis in a referral Center in Southern China. BMC Ophthalmol 2017;17(1):175.

**Figure Legends**

**Figure 1**. The preoperative and postoperative BCVA (logMAR) of PKP group.

**Figure 2**. The preoperative and postoperative BCVA (logMAR) of BB-DALK group.

**Figure 3**. **A,** Preoperative slit-lamp photograph showed a 40-year-old male patient with AK. **B,** A clear graft was observed at one year after BB-DALK. **C**, Preoperative slit-lamp photograph showed a 58-year-old female patient with AK. **D,** A clear graft was observed at one year after PKP.

**Figure 4.** Kaplan-Meier curve of graft survival rate for two groups at 1 and 3 years after surgery.

**Figure 5. A**, The HE stain of *Acanthamoeba* recurrent recipient bed in the PKP group. **B**, The *Acanthamoeba* cysts (black arrow) were detected at the cut edge of the recipient bed.

**Figure 6. A,** The HE stain of *Acanthamoeba* recurrent corneal buttons in the BB-DALK group. **B**, The *Acanthamoeba* cyst (black arrow) was detected at the graft-recipient bed junction.
